# Case of Strangulated Scrotal Hernia

**Published:** 1881-02

**Authors:** Harvey L. Byrd

**Affiliations:** Baltimore, M. D.


ARTICLE III.

### A CASE OF STRANGULATED SCROTAL HERNIA,—OPERATION, RADICAL CURE.

BY PROF. HARVÉY L. BYRD, M. D., ASSISTED BY G. HALSTED BOYLAND, A. M., M. D. OF BALTIMORE, M. D.

The small amount of space usually devoted to the consideration of strangulated scrotal hernia in some of our text books makes it all the more incumbent upon surgeons and general practitioners to report cases of this kind that may occur in their practice, and thus add to a literature that is, in this respect, at least, quite scant enough. Scrotal hernia is too apt to be regarded as simply another form of inguinal hernia (as they both pass through the internal and external ingunial rings) and thus lose some of its identity.

The following case presents several remarkable features.

James B. Trimble, living at No. 50 Fairmount Avenue, Baltimore, 30 years of age, engaged in active business, of nervo-sanguineous temperament, without any pathological history in his family, married, had been subject to scrotal relaxations in the scrotum for some years.

This relaxation was not an inguinal hernia that by long habit had descended into the scrotum, but a direct scrotal hernia, as such. It had been easily reducible, until late in the second evening before the operation; the patient usually wearing a truss. On that evening, owing to physical exertion without having on his truss, a very large quantity of intestine descended with a ruck into the scrotal sack, and symptoms of strangulation set in at once. When I first saw the case next morning, with Dr. Byrd, all attempt at reduction was out of the question. The scrotum was distended to the size of a man's head; the hernia being on the right side, and the penis quite œdematous and distorted. As Dr. Byrd assured me that he had tried every thing in the way of narcotics and anæsthetics in his efforts to accomplish postural taxis, and as the patient had been in this condition many hours already, the tumor being like a ball of lignum vitæ, as regards hardness, I agreed with him that we should operate forthwith. Chloric-ether was then administered. Dr. Byrd then made an incision over the throat of the tumor to the length of four inches, and carefully dissected the different layers of peritoneal and other tissues, and then laid open the hernial sack, when the lower constriction could be felt and seen in the shape of two fibrous bands, of the fascia transversalis, which snapped when separated by the hernial knife, and after the upper constriction was divided the reduction became a matter of no difficulty. All the protruding intestine, which was of a dark red color, just ready to go into sphacelus, was carefully replaced and the incision closed by sutures, leaving a space open at the bottom of the wound for drainage. Over the sutures were placed strips of adhesive plaster, and above all, cotton batting, wet with pyroligeneous acid and water (1 part to three). On the following day, there being no suppuration and a slight tendency to relaxation of the intestine, we put in two more sutures and closed the wound entirely, keeping up a moderate pressure by the plaster and cotton, applied as before, with an ice bag upon these. Tinct. opi. camphor. was given in teaspoonful doses, three times a day, to keep the bowels quiet. On the third day there was still no suppuration and the œdema of the penis was begining to disappear. On the fourth day the same conditions prevailed with a marked general improvement. On the fifth day, as there was abdominal uneasiness, his bowels were moved by a gentle enema and controlled with opium suppositories. From that time forth he began to convalesce and was allowed beef and eggs, his diet up to that time having consisted mainly of gruel, soups, toast, tea, etc. On the twenty-first day he sat up, and on the thirtieth left his bed, with the wound entirely healed.

We both deemed it advisable that he should wear a truss for some time, at least during the day; and he is now entirely recovered and transacting his business at his customary place.

The noticeable points in this case, are the great size of the tumor; the absence of suppuration; the upper part of the wound healing entirely by first intention, and the steady and rapid recovery after so formidable an operation.

There had been much vomiting during strangulation, but no intense prostration; only a feeling of dragging on the scrotum which caused much suffering. Doubtless our success was due to operating without delay and to the early manhood and temperate habits of the patient; and in future cases this experience will teach us to operate as soon as possible, leaving attempts at postural taxis a secondary place among surgical procedures. Dr. Byrd called attention to the fact that during the war the peritoneum was wounded in all sorts of ways, and still the patients recovered; that the intestines were wounded and in some cases a portion even removed, and still the patients got well, and suggested that we might, therefore, inflict, in the interest of science, a very slight wound, indeed, and draw off from the intestine in strangulated hernia, all liquids and gases by means of the aspirator, and thus reduce the volume of the hernia and render it, perhaps, at once reducible. The time has arrived when the *practitioner should no longer be trammeled* by the often narrow rules of text books. Authority is all very well and entitled to due respect, but experience based upon scientific truths is much better.

As to the use of the aspirator in cases of strangulated hernia, it is exceedingly doubtful if the needle prick would hurt the already compromised intestine when we bear in mind all that has been successfully practised upon the jejuneum and colon. The use of the aspirator in the intestines of the abdomen is analogous to its use in the cavities of the thorax. In aspirating in cases of pleuritic effusion, membranes are divided in much the same way and of much the same nature as those that would be penetrated in aspirating the intestine. An operation so simple, attended with but little pain, as compared with the regular operation for strangulated herniæ, ought to commend itself to the intelligent physician and surgeon. It is certain that there is always a quantity of serous fluid in the hernial sack, outside the folds of intestine; it is also certain that there is a quantity of fluid inside the intestine, varying in quality according to pathological circumstances. Who shall say then that it is not the future mission of the aspirator to withdraw both the fluids and gases, internal and external, in cases of strangulated hernia? Who shall say that it is not to render all irreducible hernia reducible, and thus supplant one of the most formidable operations in all surgery?

These suggestions of Dr. Byrd, made at the time of the operation, are as much for ourselves as for others. We offer them with the fixed intention of putting them to a practical application the first occasion permitting itself and of giving to science the benefit of our experience. Not yet having seen recorded a single case of strangulated hernia in which the aspirator has been used in this particular way, and being very much interested in the subject, we trust we shall be pardoned for having trespassed thus far upon your patience, especially when there is in it a possible way of superseding the knife and of saving human life.

